# *In Vivo* Pharmacological Evaluations of Novel Olanzapine Analogues in Rats: A Potential New Avenue for the Treatment of Schizophrenia

**DOI:** 10.1371/journal.pone.0080979

**Published:** 2013-12-11

**Authors:** Somayeh Jafari, Xu-Feng Huang, Jessica L. Andrews, Francesca Fernandez-Enright

**Affiliations:** 1 Illawarra Health and Medical Research Institute and School of Medicine, University of Wollongong, Wollongong, New South Wales, Australia; 2 School of Chemistry, University of Wollongong, Wollongong, New South Wales, Australia; 3 Schizophrenia Research Institute, Darlinghurst, New South Wales, Australia; University of Santiago de Compostela School of Medicine - CIMUS, Spain

## Abstract

Olanzapine (Olz) is one of the most effective antipsychotic drugs commonly used for treating schizophrenia. Unfortunately, Olz administration is associated with severe weight gain and metabolic disturbances. Both patients and clinicians are highly interested in the development of new antipsychotics which are as effective as atypical antipsychotics but which have a lower propensity to induce metabolic side effects. In the present study, we examined two new derivatives of Olz; OlzEt (2-ethyl-4-(4′-methylpiperazin-1′-yl)-10*H*benzo[*b*]thieno[2,3-*e*][1,4]diazepine), and OlzHomo (2-ethyl-4-(4′-methyl-1′,4′-diazepan-1′-yl)-10*H*-benzo[*b*]thieno[2,3-*e*] [1,4]diazepine), for their tendency to induce weight gain in rats. Weight gain and metabolic changes were measured in female Sprague Dawley rats. Animals were treated orally with Olz, OlzEt, OlzHomo (3 or 6 mg/kg/day), or vehicle (n = 8), three times daily at eight-hour intervals for 5 weeks. Furthermore, a phencyclidine (PCP)-treated rat model was used to examine the prevention of PCP-induced hyperlocomotor activity relevant for schizophrenia therapy. Male Sprague Dawley rats were pre-treated with a single dose (3 mg/kg/day) of Olz, OlzEt, OlzHomo, or vehicle (n = 12), for 2 weeks. Locomotor activity was recorded following a subcutaneous injection with either saline or PCP (10 mg/kg). Olz was found to induce weight gain, hyperphagia, visceral fat accumulation, and metabolic changes associated with reduced histamatergic H_1_ receptor density in the hypothalamus of treated rats. In contrast, OlzEt and OlzHomo presented promising antipsychotic effects, which did not induce weight gain or fat deposition in the treated animals. Behavioural analysis showed OlzEt to attenuate PCP-induced hyperactivity to a level similar to that of Olz; however, OlzHomo showed a lower propensity to inhibit these stereotyped behaviours. Our data suggest that the therapeutic effectiveness of OlzHomo may be delivered at a higher dose than that of Olz and OlzEt. Overall, OlzEt and OlzHomo may offer a better pharmacological profile than Olz for treating patients with schizophrenia. Clinical trials are needed to test this hypothesis.

## Introduction

Olanzapine (Olz) is an effective atypical antipsychotic drug used for treating severe psychiatric disorders, including schizophrenia and bipolar disorder [1], [2]. However, Olz administration is associated with excessive weight gain and severe metabolic side effects such as type II diabetes mellitus, hyperglycemia, dyslipidemia, and insulin resistance [1], [3], [4], [5], [6]. A novel antipsychotic drug that retains the clinical efficacy of Olz but causes less treatment-emergent weight gain would be an invaluable breakthrough in schizophrenia therapy. For the last few years, researchers have focused on developing novel antipsychotics with fewer metabolic side effects [7]. Nonetheless, the crucial need for developing an ideal antipsychotic agent for schizophrenia still continues. It is suggested that the simultaneous blockade of the dopamine D_2_ and serotonin 5HT_2A_ receptors with Olz through the various dopaminergic pathways is involved in the molecular mechanisms of Olz's therapeutic efficacy, resulting in the distinct clinical properties of this drug [8], [9], [10], [11]. Changes in hormonal peptide levels correlated with food intake (such as insulin and leptin) have been suggested to play a part in the weight gain induced by atypical antipsychotic drug treatments [12]. Olz in particular significantly influences the regulation of plasmatic insulin and leptin, although the mechanism of action remains elusive [13], [14]. A large number of studies have also reported a relevant role for the affinities of atypical antipsychotics for the serotonergic 5HT_2A_, 5HT_6_, and 5HT_7_ receptors; adrenergic α_1A_ receptor; and particularly histamatergic H_1_ and serotonergic 5HT_2C_ receptors in their obesogenic effects [2], [15], [16], [17].

The blockade of the H_1_ receptors has been repeatedly described as the most likely mechanism for atypical antipsychotic drug-induced weight gain [18], [19], [20]. The inhibition of the H_1_ receptors is directly involved in the activation of hypothalamic AMPK (5′ adenosine monophosphate-activated protein kinase) signalling, which stimulates food intake and positive energy balance and reverses the anorexigenic effects of leptin [18]. A strong link between H_1_ receptor affinity with antipsychotic agents and weight gain susceptibility has been reported [21]. Clozapine and Olz, which have a higher affinity for the H_1_ receptors (*K*_i_ = 1.2 nM and *K*_i_ = 2.0 nM, respectively), showed a greater propensity to induce weight gain [15], [22]. However, antipsychotic drugs with a lower H_1_ receptor antagonist affinity, such as loxapine and amoxapine, caused neither weight gain nor weight loss in patients treated with these medications [23], [24]. Thus, the development of a novel antipsychotic agent with a similar 5HT_2A_/D_2_ receptor binding affinity ratio to that of Olz [25], [26], and with a lower affinity for the H_1_ receptors, may significantly advance schizophrenia therapy.

We have previously examined two new analogues of Olz; OlzEt (2-ethyl-4-(4′-methylpiperazin-1′-yl)-10*H*benzo[*b*]thieno[2,3-*e*][1], [4]diazepine) [68], and OlzHomo (2-ethyl-4-(4′-methyl-1′,4′-diazepan-1′-yl)-10*H*-benzo[*b*]thieno[2,3-*e*] [1,4]diazepine) (newly synthesised by our research group) [27], presenting an ethyl substituent at position 2 of the thiophene ring of Olz compounds. We have demonstrated in our previous published study [27] that both of these analogues showed an *in vitro* lower affinity for the H_1_ receptors while maintaining similar affinities for the D_2_ and 5HT_2A_ receptors when compared to Olz. Since the blockade of the H_1_ receptors has been repeatedly described as the most likely mechanism for atypical antipsychotic drug-induced weight gain, the present study further explored the therapeutic potential *in vivo* of these two analogues of Olz in an animal model of schizophrenia while assessing their metabolic side effects. We found that OlzEt and OlzHomo compounds display a significant reduction in metabolic side effects (weight gain and adiposity) compared to Olz. Thus OlzEt and OlzHomo may present as potential new drugs for schizophrenia therapy.

## Materials and Methods

### Ethics statement

All experimental procedures were approved by the Animal Ethics Committee, University of Wollongong, and conducted in accordance with the *Australian Code of Practice for the Care and Use of Animals for Scientific Purposes* (2004).

#### Study (1) Animals and drug treatment regimes

In the first series of experiments (Study 1), female Sprague Dawley rats (7 weeks old) were used to investigate weight gain and adiposity effects of a chronic treatment with Olz, OlzEt, and OlzHomo. Animals were obtained from the Animal Resources Centre (Perth, WA, Australia) and housed individually at 22°C, on a 12 h light-dark cycle with *ad libitum* access to water and standard laboratory chow diet (3.9 kcal/g, 74% carbohydrate, 16% protein, and10% fat). Animals were then randomly assigned to one of the following treatment groups: 3 or 6 mg/kg/day of Olz (Bosche Scientific, NJ, USA), OlzEt (Lichem, Hebei Boyuan Co., China), OlzHomo (Lichem, Hebei Boyuan Co., China), or vehicle (n = 8), three times daily at eight-hour intervals. Following 1 week habituation in their new environment, the animals underwent training to self-administer a sweet cookie dough pellet for 1 week. Cookie dough (62% carbohydrate, 22% protein, 10% vitamins, 6% fiber, and minerals) administration was performed as previously reported for 5 weeks [33]. Over the course of this experiment, animals were weighed twice per week. Food and water consumption were also monitored every 48 hours for each animal and results were corrected for spillage.

#### Post-mortem hormone, lipid and tissue analysis

At the end of Study 1, female rats were fasted for 10 h prior to sacrifice by carbon dioxide asphyxiation. Upon sedation, blood was removed and collected in Lavender Vacutainer tubes containing EDTA (ethylenediaminetetraacetic acid; 5-HT, 5-hydroxytryptamine (serotonin) for hormonal testing. Samples were immediately centrifuged (1000 g for 10 min at 4°C), after which plasma was aliquoted and stored at −20°C until use. Fasting plasma insulin, leptin, and adiponectin levels were measured using commercially available Milliplex kits (Millipore Corp., USA) and Luminex 100. Plasma samples were processed by Southern IML Pathology for levels of glucose, cholesterol, triglycerides, high-density lipoprotein (HDL), and low-density lipoprotein (LDL) levels. White fat pads and sub-scapula brown fat pads were dissected from each animal and individually weighed. Brains were immediately removed, dissected into hypothalamus and prefrontal cortex, snap frozen in liquid nitrogen and then stored at -80°C until use.

#### Study (2) Animals and drug treatment regimes

In the next experiment (Study 2), the effects of Olz, OlzEt, and OlzHomo subchronic administration on PCP-induced behaviours were tested in male Sprague Dawley rats (180–200 g). Animals were housed in pairs in the same conditions described above. Following a 1 week habituation period, rats were treated orally with a sweet cookie dough pellet containing 3 mg/kg/day of Olz, OlzEt, OlzHomo, or vehicle (n = 12), three times daily at eight-hour intervals for 2 weeks. Animals were injected subcutaneously with either saline or PCP (10 mg/kg, synthesized in the School of Chemistry, University of Wollongong, Wollongong, New South Wales, Australia) 30 min following the final drug/cookie administration. Open-field behavioural testing was performed 15 min after this injection.

#### Behavioural analysis and post mortem measurement

The open field test was used to determine the behavioural effects of the pre-treatment of Olz, OlzEt, and OlzHomo on PCP-treated animals. To minimise stress during the experiment, animals underwent a 10 min habituation period for the open-field test one day prior to the experiment. As previously described [65], the locomotor activity was recorded for each tested animal in a black open square box (60 cm×60 cm×40 cm). Behavioural parameters including the total distance travelled (cm), mean velocity (cm/s), central and peripheral duration (s), and frequency of rearing were measured for 30 min and then analysed *via* Ethovision video-tracking software (Nodulus Information Technology, Wageningen, The Netherlands). Animals were euthanized 120 min following the open-field test as described above; brains were rapidly removed from the skull and dissected into prefrontal cortex and striatum, snap frozen in liquid nitrogen and stored at −80°C until required for the receptor binding assays.

#### Radioligand binding assays

The striatum, prefrontal cortex, and hypothalamus were used in radioligand binding assays to measure the receptor binding density of D_2_, 5HT_2A_, and H_1_ receptors respectively. The assays were performed according to previously described procedures [66], [67]. In brief, the striatum, prefrontal cortex, and hypothalamus were homogenized separately and then centrifuged (27,000 g for 15 min at 4°C). The resultant membrane was incubated in the presence of 2 nM [^3^H]-Spiperone (specific activity, 15 Ci/mmol, 1mCi/ml; Perkin Elmer, Australia), with or without 2 µM (+) butaclamol (Sigma, Australia), 10 nM [^3^H]-Ketanserine (specific activity, 67 Ci/mmol, 1mCi/ml; Perkin Elmer, Australia) in the absence or presence of 10 µM methysergid (Sigma, Australia), or Pyrilamine (specific activity, 37 Ci/mmol, 1mCi/ml; Perkin Elmer, Australia) with or without 2 µM doxepin (Sigma, Australia), for D_2_, 5HT_2A_, and H_1_ receptor binding assays, respectively. Radioactivity was measured by a beta liquid scintillation analyser (Perkin Elmer, Tri-Crab 2800 TR).

#### Data analysis

Data were statistically analysed using SPSS (version 17.0 SPSS, Chicago, IL, USA). Total weight gain, total food intake, energy efficiency, insulin, leptin, adiponectin, glucose, cholesterol, triglycerides, HDL, LDL, fat mass, and binding density were analysed by one-way analyses of variance (ANOVA) for each dose of Olz, OlzEt, and OlzHomo. Repeated ANOVA measures (COMPOUNDS×DAYS as repeated measures) were employed for cumulative weight gain, and food and water intake. Open-field parameters were also analysed by one-way ANOVA. Student's t-tests were used to determine the significance of differences between the saline and PCP-treated rats. Multiple comparisons were performed using Tukey or Games–Howell post hoc tests. Where Kolmogorov–Smirnov tests showed data to be distributed non-parametrically, Kruskall–Wallis tests were applied followed by Mann–Whittney U post hoc analysis. Correlations were identified using Pearson's correlation tests or Spearman's correlation tests for non-parametric data. Linear regression was performed in groups with significant correlations. Significance was set at P<0.05.

## Results

### Study (1) Weight gain and metabolic side effects of Olz, OlzEt, and OlzHomo

#### Body weight gain, food and water intake

**Figure 1:**
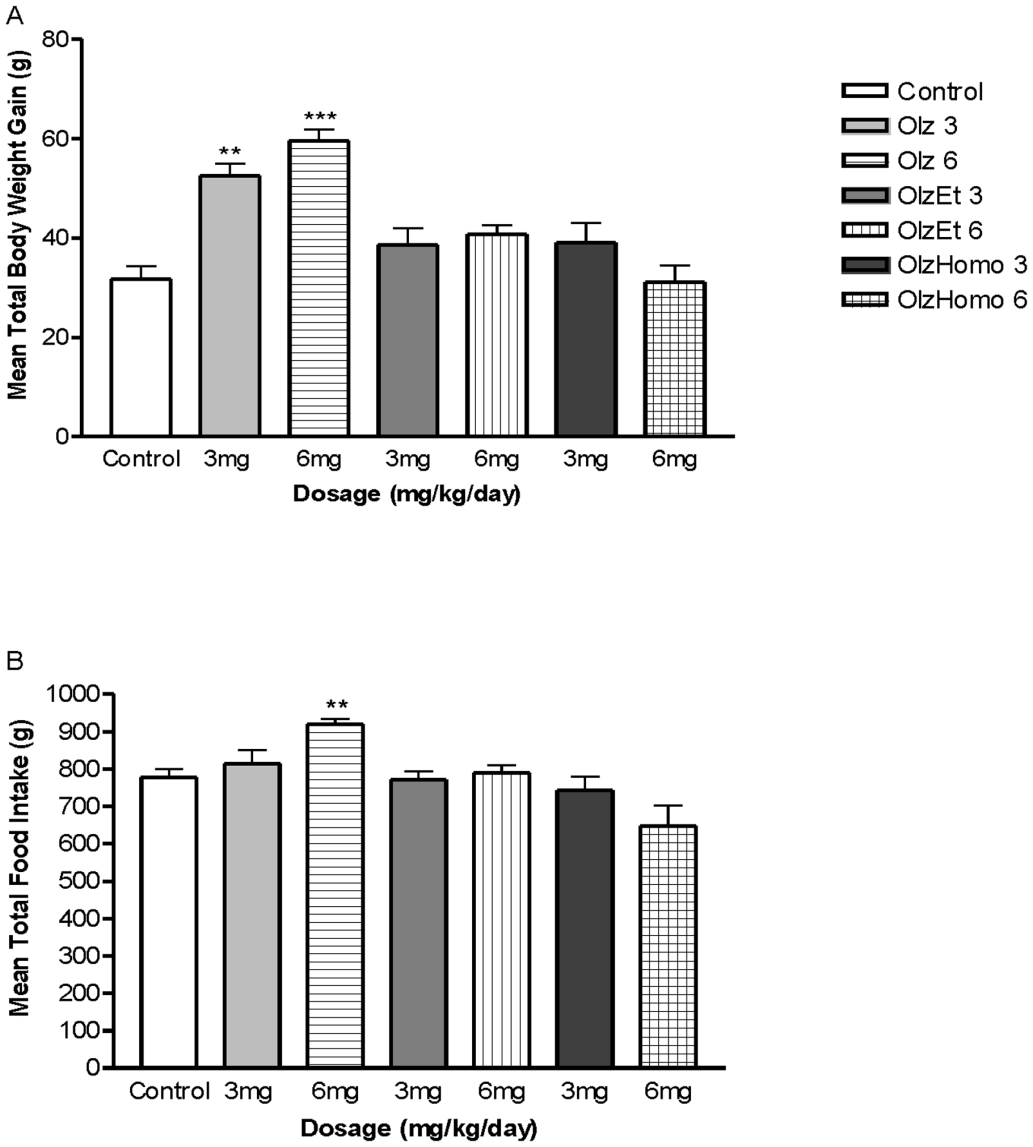
Total weight gain and food intake in female rats treated with Olz and Olz derivatives. A. Total body weight gain (g), B. Total food intake (g) in female Sprague Dawley rats treated with Olz, OlzEt, OlzHomo (3 mg/kg or 6 mg/kg), or vehicle (Control) for 5 weeks. The data points are the mean ± SEM, **P<0.01 and ***P<0.001 *vs*. Control.

**Figure 2:**
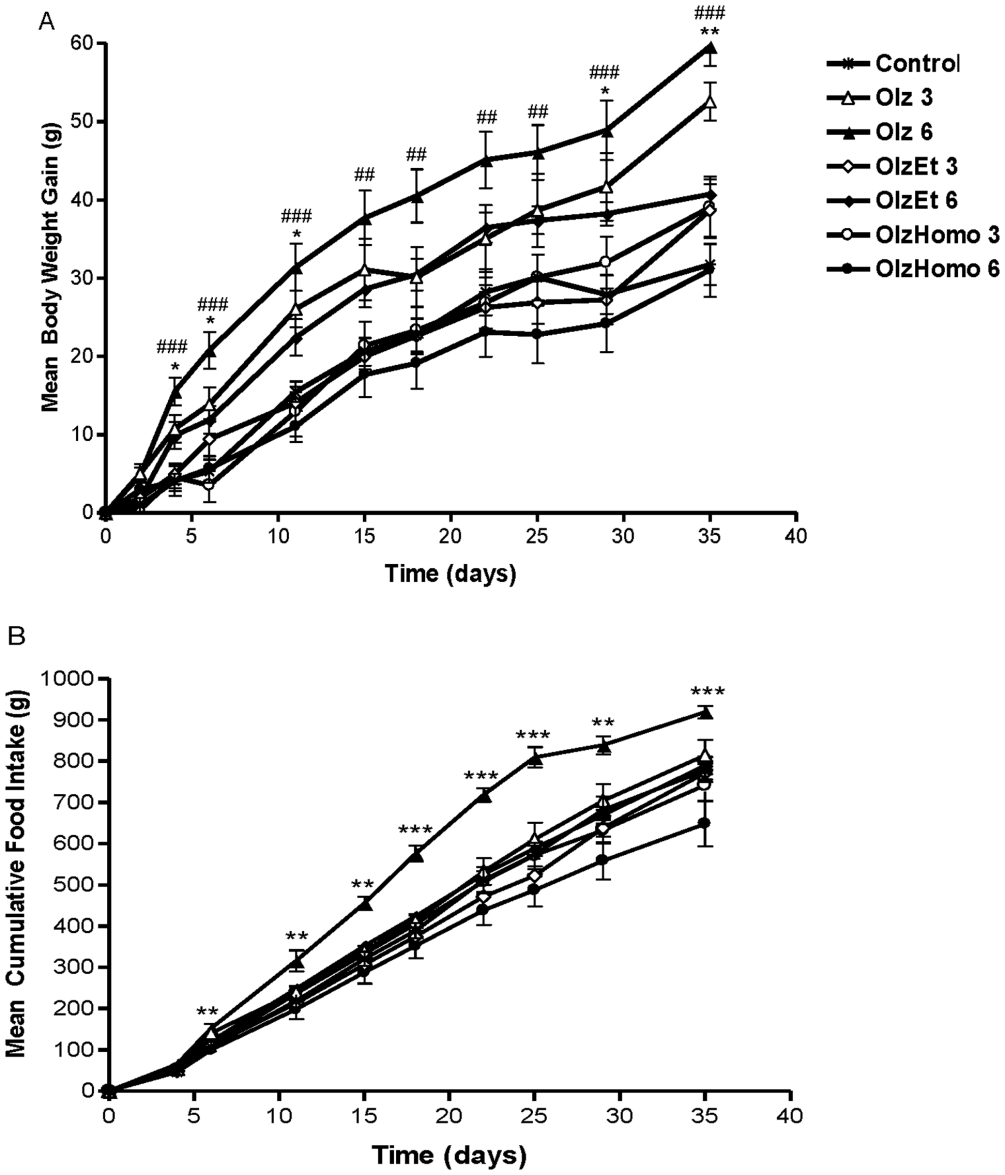
Cumulative weight gain and food intake in female rats treated with Olz and Olz derivatives. A. Cumulative body weight gain (g), B. Cumulative food intake (g) in female Sprague Dawley rats treated with Olz, OlzEt, OlzHomo (3 mg/kg or 6 mg/kg), or vehicle (Control) for 5 weeks. The data points are the mean ± SEM. A. *P<0.05, **P<0.01: Olz 3 mg/kg *vs.* control. ^##^ *P*<0.01, ^###^ *P*<0.001: Olz 6 mg/kg *vs*. Control. B. **P<0.01, ***P<0.001: Olz 6 mg/kg *vs*. Control. (*: Control, Δ: Olz 3 mg/kg, ▴: 6 mg/kg, ◊: OlzEt 3 mg/kg, ♦: OlzEt 6 mg/kg, o: OlzHomo 3 mg/kg and •: OlzHomo 6 mg/kg).

Body weight gain was found to be significantly increased following both 3 mg/kg (F_3,27_ = 7.11, P = 0.001) and 6 mg/kg (F_3,27_ = 23.27, P<0.001) Olz administration compared to the control group. However, the effects of both OlzEt and OlzHomo on body weight gain were not significantly altered compared to the control groups. OlzEt and OlzHomo showed a significant reduction in weight gain compared to the Olz group with 3 mg/kg (−26%, P<0.05 for both compounds) and 6 mg/kg doses (−32% and −48%, P<0.001, respectively) (Figure 1). A repeated ANOVA measure (treatment×days) revealed a significant effect of time on the progressive enhancement of body weight for both doses of Olz (3 mg/kg and 6 mg/kg) (F_3.37,90.90_ = 246.06, P<0.001, and F_3.32,92.89_ = 289.38, P<0.001, respectively), and the interaction between these two factors (F_10.10,90.90_ = 2.34, P<0.05, and F_9.95,92.89_ = 5.53, P<0.001, respectively). As illustrated in Figure 2, administration of Olz (at both tested doses) gradually increased weight gain from day 4 to the end of the treatment period. In contrast, the effect of OlzEt and OlzHomo on cumulative body weight was not significant compared to controls (Figure 2). Higher doses of Olz treatment (6 mg/kg) induced a significant increase in food intake (F_3,27_ = 10.73, P = 0.001) that began after 6 days of treatment. In contrast, OlzEt and OlzHomo administration did not affect food intake for either of the tested doses. Post hoc analysis showed that total food intake after 5 weeks of treatment with OlzEt and OlzHomo (6 mg/kg) was significantly lower than Olz administration (14% and P = 0.001, 29% and P = 0.006, respectively) (Figure 2)). A significant positive correlation between total body weight gain and total food intake was found after 5 weeks of treatment (r = 0.48, P<0.001) (Table S1).

#### Fat deposition

Visceral fat deposition (intra-abdominal, including retroperitoneal) was significantly increased in 6 mg/kg Olz treated rats compared to the control group (*F*_3,28_ = 8.98, *P* = 0.002). However, OlzEt and OlzHomo treated animals (3 mg/kg and 6 mg/kg, *P*>0.05) did not show any significant difference in relation to visceral fat deposition compared to controls (Table 1). Significant positive correlations were found between body weight gain and food intake with fat mass (*r* = 0.42, *P* = 0.002 and *r* = 0.59, *P*<0.001, respectively) (Table S1). There were no effects of treatments on sub-scapula brown fat mass compared to the control group (Table 1).

**Table 1:** Body weight and fat pad mass in female rats treated with Olz and Olz derivatives.

	Control	Olz		OlzEt		OlzHomo	
		3 mg	6 mg	3 mg	6 mg	3 mg	6 mg
*Body Weight (g)*							
IBW	226.43±10.74	225.38±10.04	225.18±8.79	228.09±8.46	224.68±9.57	226.00±9.8	223.61±9.5
FBW	252.46±11.01	281.08±12.14	**290.38±7.16** *	266.75±9.8	265.40±10.00	265.038±7.7	253.7±10.00
*Fat pad mass (g)*							
Total White fat	6.31±0.93	8.01±1.01	**12.18±1.14** ***	6.32±0.73	8.57±1.12	6.01±1.06	5.17±0.91
Subscapula (BAT)	0.32±0.04	0.30±0.04	0.27±0.3	0.27±0.2	0.24±0.2	0.27±0.02	0.25±0.2
Total white fat/FBW (%; g/g)	2.36±0.29	2.85±0.28	**4.28±0.31** ***	2.32±0.19	3.16±0.30	2.22±0.35	1.96±0.27

± SEM, Mean body weight and fat pad mass in female Sprague Dawley rats following 5 weeks treatment with Olz, OlzEt, OlzHomo (3 mg/kg or 6 mg/kg), or vehicle (Control). IBW: initial body weight, FBW: final body weight. Data are expressed as mean

*P*<0.05, and.

*** *P*<0.001 *vs*. Control.

#### Plasma hormone and glucose levels

**Figure 3:**
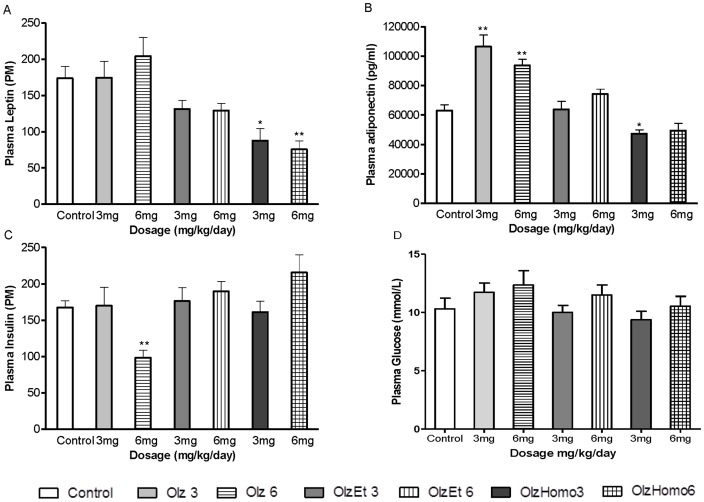
Hormonal profiles in female rats treated with Olz and Olz derivatives. A. Plasma Leptin (PM), B. Plasma Adiponectin (pg/ml), C. Plasma insulin (PM), D. Plasma Glucose (mmol/L) in female Sprague Dawley rats treated with Olz, OlzEt, OlzHomo (3 mg/kg or 6 mg/kg), or vehicle (Control) for 5 weeks. The data points are the mean ± SEM, *P<0.05 and **P<0.01 *vs*. Control.

Olz treatment at a dose of 6 mg/kg caused a significant reduction in fasting plasma insulin levels in the tested animals compared to the control rats (*F*_3,23_ = 10.46, *P*<0.01). In contrast, OlzEt and OlzHomo administration did not affect the fasting plasma insulin levels in the tested rats compared to the controls. Additionally, both compounds showed higher insulin levels than those measured in Olz treated animals with 6 mg/kg doses (48%, *P* = 0.001, and 54%, *P* = 0.013, respectively) (Figure 3). The insulin levels were not significantly altered in the treated groups (Olz, OlzEt, and OlzHomo) for the 3 mg/kg dose compared to the control animals. Post hoc analysis did not show any significant difference in the leptin levels of animals treated with Olz at both 6 mg/kg (*F*_3,22_ = 6.92, *P* = 0.95) and 3 mg/kg (*F*_3,21_ = 5.33, *P = *1.00) doses compared to the control groups. However, plasma leptin levels were significantly decreased following both 3 mg/kg and 6 mg/kg OlzHomo (*P = *0.01 and *P*<0.01, respectively) compared to controls. Fasting plasma adiponectin levels were found to be significantly higher after chronic administration of Olz (at the 3 mg/kg and 6 mg/kg doses, *F*_3,23_ = 22.45, *P*<0.01, and *F*_3,24_ = 18.28, *P*<0.001, respectively) but were found to be lower following OlzHomo treatment (at the 3 mg/kg dose, *P* = 0.039) compared to controls (Figure 3). Both leptin and adiponectin levels for the animals treated with OlzEt were not significantly altered at both tested doses compared to controls) (Figure 3). A significant negative correlation was found between plasma insulin levels and food intake (*r* = −0.46, *P* = 0.002), as well as a significant positive correlation between leptin levels and fat deposition (*r* = 0.34, *P* = 0.026) (Table S1). Plasma glucose levels were found to be not significantly altered following treatment with Olz, OlzEt or OlzHomo at either the 3 mg/kg dose or the 6 mg/kg dose compared to the control rats (Figure 3).

#### Plasma lipid levels

Levels of plasma cholesterol, triglycerides and LDL were found to be unaltered in the treatment groups (Olz, OlzEt, OlzHomo) at both the 3 mg/kg and the 6 mg/kg dose compared to controls (Table 4). HDL levels were found to be significantly decreased in both the OlzEt 6 mg/kg treated animals and in the OlzHomo 3 mg/kg treated animals compared to controls (P = 0.027 and P = 0.007 respectively) (Table 4). However there was no significant difference in HDL levels in any of the other treatment groups at either dose (Table 4).

**Table 4:** Plasma lipid and glucose profiles in female rats treated with Olz and Olz derivatives.

	Control	Olz		OlzEt		OlzHomo	
		3 mg	6 mg	3 mg	6 mg	3 mg	6 mg
Cholesterol (mmol/L)	1.98±0.22	1.50±0.09	1.81±0.08	1.63±0.08	1.63±0.06	1.75±0.07	1.50±0.06
Triglycerides (mmol/L)	1.12±0.13	0.85±0.12	1.11±0.13	0.89±0.12	0.83±0.13	0.90±0.08	0.88±0.07
HDL (mmol/L)	0.18±0.01	0.14±0.02	0.16±0.02	0.12±0.01	0.17±0.02*	0.20±0.03**	0.13±0.01
Cholesterol/HDL ratio (mmol/L)	11.55±1.26	12.03±1.58	12.08±1.36	13.30±0.68	10.81±1.61	10.50±1.37	12.81±1.32
LDL (mmol/L)	1.30±0.18	0.98±0.09	1.14±0.06	1.09±0.08	1.09±0.02	1.14±0.07	0.86±0.12

Mean concentrations of plasma lipids and glucose in female Sprague Dawley rats following 5 weeks treatment with Olz, OlzEt, OlzHomo (3 mg/kg or 6 mg/kg), or vehicle (Control). Data are expressed as mean ± SEM,

* *P*<0.05, and.

** *P*<0.01 *vs*. Control.

#### Aterations in H_1_ receptor density

Olz (3 mg/kg and 6 mg/kg) significantly reduced the H_1_ receptor density in the hypothalamus of the female animals treated for 5 weeks (F_3,12_ = 4.01, P<0.05, and F_3,12_ = 23.06, P<0.001, respectively) compared to controls, while the H_1_ receptor density remained unchanged in animals treated with either OlzEt or OlzHomo for both tested doses (Table 2). There was a significant negative correlation between total body weight gain and visceral fat mass with H_1_ receptor density in the hypothalamus (r = −0.59, P = 0.001, and r = −0.61, P = 0.001, respectively) (Table S1).

**Table 2:** Brain receptor densities in female rats treated with Olz and Olz derivatives.

Receptor	Control	Olz		OlzEt		OlzHomo	
		3 mg	6 mg	3 mg	6 mg	3 mg	6 mg
H_1_	365.2±15.9	289.7±4.6*	**209.2±14.9** ***	344.2±26.9	367.2±22.4	351.2±9.5	360±7.7
D_2_							
*Saline*	2009.2±27.9	**1677±23.6** ***		1938.2±18.6		**1826±26.9** ***	
*PCP*	2031.8±42.9	**1403.2±68.6** **		**1651.7±41.2** **		2088.5±45.9	
5HT_2A_							
*Saline*	7391±264.8	**2919±76.3** ***		**5528±117.9** **		6604.2±178.2	
*PCP*	8835.8±187	**2742±125.8** ***		**5682±76.3** ***		8421.2±537.9	

H_1_ receptor specific density in the hypothalamus of female Sprague Dawley rats following 5 weeks treatment with Olz, OlzEt, OlzHomo (3 mg/kg or 6 mg/kg), or vehicle (Control). D_2_, and 5HT_2A_ receptor specific densities in the striatum and prefrontal cortex of male Sprague Dawley rats, respectively, following 2 weeks pre-treatment with Olz, OlzEt, OlzHomo (3 mg/kg), or vehicle (Control) and subcutaneous injection of Saline or PCP. Data are expressed as CPM: counts per minute; mean ± SEM,

** *P*<0.01, and.

*** *P*<0.001 *vs*. Control.

### Study (2) Behavioural and neurochemical study: A comparison between Olz, OlzEt, and OlzHomo treatments

#### Behavioural testing

Behavioural results are illustrated in Table 3. In the saline groups, no significant difference was found following 2 weeks of treatment with Olz, OlzEt, or OlzHomo in all the parameters measured in the open-field test. However, in animals acutely administered with PCP, total distance moved, mean velocity, and centre and periphery durations differed significantly according to the tested treatments (Olz, OlzEt, and OlzHomo). No sedative behaviour was observed in PCP-treated rats. Total distance moved was significantly reduced in Olz and OlzEt (F_3,31_ = 4.97, P = 0.023, and F_3,31_ = 8.19, P = 0.008) treated rats compared to controls. However, the effect of OlzHomo treatment on reducing locomotor activity was not significant compared to the controls (P = 0.42). Mean velocity was also reduced in animals treated with Olz (F_3,44_ = 8.22 and P<0.05). One-way ANOVA revealed significant changes in the centre and periphery durations following 2 weeks of treatment with OlzEt (F_3,33_ = 4.37, P<0.01, and F_3,41_ = 3.21, P = 0.017, respectively). Moreover, t-test results showed a significant effect of acute PCP administration on the distance travelled in animals treated with Olz, OlzEt, OlzHomo or control compared to their equivalent saline groups.

**Table 3:** Open field test in male rats treated with Olz and Olz derivatives.

Open field test	Control	Olz	OlzEt	OlzHomo
*Saline*				
Total distance moved (%)^a^	135.5±36.6	110.2±31.9	143.1±49.1	118.7±33.1
Mean velocity (cm/s)	20.2±3.7	18.2±3.3	14.0±1.0	16.9±1.8
Rearing frequency (%)^a^	118.6±19.5	109.4±30.5	122.7±23.1	116.9±22.1
Centre duration (s)	40.68±10.1	40.34±6.4	51.52±93	86.16±28.9
Periphery duration	1820.5±17.5	1827.8±28.3	1792.0±16.2	1818.3±14.6
Centre frequency	22.2±2.7	18.5±2.5	23.6±2.8	23.7±3.6
Periphery frequency (%)^a^	106.5±28.9	133.0±39.0	132.5±20.8	118.3±63.4
*PCP*				
Total distance moved (%)^a^	357.6±33.5##	**217.9**±39.6* #	**198.1**±28.9**	280.8±26.6###
Mean velocity (cm/s)	22.0±1.5#	**11.7**±2.1* #	15.1±2.9	26.4±2.4#
Rearing frequency (%)^a^	209.6±40.0	233.3±71.0	258.6±69.6	176.8±29.8
Centre duration (s)	101.2±19.5##	234.4±62.9#	**281.0**±36.11** ###	127.6±38.4
Periphery duration	1770.5±24.8	1666.6±93.6	**1585.4**±48.7* ###	1768.0±36.7
Centre frequency	47.1±4.9###	36.5±6.1#	54.3±9.2###	45±11.8
Periphery frequency (%)^a^	245.6±24##	285.9±99.6	376.4±54.8##	238.7±53.3

Open field testing in male Sprague Dawley rats following 2 weeks treatment with Olz, OlzEt , OlzHomo (3 mg/kg), or vehicle (Control). Data are expressed as mean ± SEM,

* *P*<0.05 and.

** *P*<0.01 *vs*. Control.

# *P*<0.05,

## *P*<0.01, and.

### *P*<0.001 *vs*. Saline.

a Normalised data.

#### Alteration in D_2_ and 5HT_2A_ receptor densities

In the saline groups, 2 weeks of treatment with Olz and OlzHomo induced a significant reduction in D_2_ receptor density in the striatum of the male rats compared to controls (F_3,20_ = 34.83, P<0.001, and F_3,20_ = 31.25, P<0.001, respectively). However, in the PCP-treated groups, a significant reduction was found following Olz and OlzEt treatments (F_3,20_ = 40.75, P = 0.005) compared to controls. Similarly, 5HT_2A_ receptor density in the prefrontal cortex of the animals treated with Olz and OlzEt was significantly decreased in both saline (F_3,20_ = 125.03, P<0.001, and F_3,20_ = 125.03P = 0.002, respectively) and PCP (F_3,20_ = 92.13, P<0.001) groups (Table 2).

## Discussion

### Pharmacological and behavioural evidence of effective analogues of Olz in an animal model: possible applications in the clinic

N-methyl-D-aspartate (NMDA) receptor antagonist animal models of schizophrenia such as PCP have been widely used to test new drugs that have been developed for future schizophrenia therapies [53]. These models offer reasonable validity with respect to the clinical symptoms of schizophrenia, and to some degree predict the efficacy of drugs in patients. In our study, we used the PCP rat model to determine whether sub-chronic pre-treatment with Olz, OlzEt, or OlzHomo could attenuate the characteristic PCP-induced behaviours in adult male rats. We chose to focus this study specifically in male rats since the effects of PCP injections have been shown to be more pronounced in male compared to female rats in several behavioural tests [69], [70]. Coinciding with previous reports [54], [55], [56], our results showed that PCP-treated rats showed a remarkable increase in spontaneous locomotor activity (i.e. total distance travelled or travel velocity) and anxiety/exploratory related parameters (duration/frequency in centre or periphery) of the open-field test compared to the saline group. It has been suggested that the behavioural effects of PCP treatment are due to the multiple mechanisms of action that may include altered dopamine, serotonin and noradrenaline transmission [53], [57], [58]. For instance, a disruption to the firing pattern of dopaminergic neurons, which increases dopamine release in the frontal cortex and activates the mesolimbic dopaminergic neurons, may play a part in the PCP-induced psychotic behaviour which is similar to that seen in schizophrenia patients. Altered activity of glutamatergic neurons in the cortex, leading to elevated glutamate release and a reduced inhibitory feedback onto the principal neurons, is also involved in this mechanism [53].

Regarding its clinical relevance, Olz has been shown to attenuate the hyperlocomotion and anxiety induced by PCP administration in animals [59]. In our study, the observed PCP-induced behaviours were largely blocked in the Olz and OlzEt treatments in rats. Pre-treatment with Olz and OlzEt significantly inhibited the hyperlocomotion induced by PCP in male rats. Interestingly, OlzEt was more effective than the Olz treatment in suppressing the anxiety-like behaviours of PCP, such as longer time spent in the outer field and lower entries into the centre field, suggesting the potential antipsychotic capacity of OlzEt. In contrast, OlzHomo did not suppress the PCP-induced behaviours measured in the open-field test. It is suggested that the mixed antagonistic activity of Olz at multiple receptors, including dopamine D_1_–D_4_; serotonin 5HT_2A_ and 5HT_2C_; muscarinic M_1_; and adrenergic α_1_, and α_2_ receptors, may underlie its ability to block PCP-induced behaviours [53], [60]. For instance, an increased level of serotonin at synapses containing 5HT_2A_ receptors plays a part in PCP-induced hyperlocomotion. This effect may be prevented by 5HT_2A_ antagonism by Olz at the level of motor pathways in the spinal cord or in the brain [61]. In our previous study [27], OlzEt showed a similar affinity as Olz for blocking the D_2_ and 5HT_2A_ receptors in the striatum and prefrontal cortex respectively, which may partly explain the way in which OlzEt inhibits the PCP-induced behaviours *in vivo*. On the other hand, OlzHomo demonstrated a lower affinity for blocking these two receptors, which may contribute to its lack of efficacy for alleviating the PCP-induced hyperactivity. In fact, since the ambulation in OlzHomo/PCP-treated rats was comparable to the control/PCP group, we postulate that the potential for therapeutic effectiveness of an OlzHomo regime may be achieved at a higher dose than that at which Olz and OlzEt are administered.

To further validate our hypothesis regarding the effect of PCP on the levels of D_2_ and 5HT_2A_ receptors, we measured the neurochemical changes in the brain following the PCP challenge. Consistent with previous reports [51], [62], [63], our findings showed that the D_2_ and 5HT_2A_ receptor densities in the striatum and prefrontal cortex respectively, in adult male PCP-treated rats, did not differ from saline-treated controls. However, subchronic treatment with Olz and OlzEt induced a long-lasting down-regulation in the binding capacities of D_2_ and 5HT_2A_ receptors in both saline and PCP-treated animals. As previously suggested, the down-regulation of these two receptors may partly contribute to the blockade of PCP-induced behavioural changes, including hyperlocomotion [61], [64]. However, this hypothesis should be taken with a degree of caution, since altered dopamine and serotonin receptor densities are not the only mechanisms underlying the behavioural changes induced by PCP administration [53]. The extent to which such changes are involved in the therapeutic effects of Olz and OlzEt remains to be investigated. However, based on the pharmacological and behavioural results reported in our study, OlzEt warrants further examination for the treatment of schizophrenia.

### Prevention of weight gain and adiposity following OlzEt and OlzHomo regimes

In contrast with study 2, female rats were used in study 1 since the metabolic side effects following antipsychotic treatment (such as Olz) reported in the literature have been more pronounced in females compared to males [72]. The switch of gender for this study should not influence the results since the most appropriate animal model was chosen to be able to validate the different hypothesis in the two respective studies. Similar to previous animal studies [28] and clinical reports [29], our study showed that chronic treatment with Olz (at both 3 and 6 mg/kg doses) induced an increase in body weight gain, with higher doses causing a greater effect. In contrast OlzEt, and particularly OlzHomo, prevented weight gain over the treatment period. Our study also confirmed previous findings in relation to hyperphagia and enhanced energy efficiency induced by Olz treatment [30], [31], [32]. Notably, food intake was increased in animals treated with 6 mg/kg of Olz, indicating that energy consumption contributed significantly to body weight gain in this group of animals. On the other hand, food intake was not significantly increased in the 3 mg/kg Olz-treated rats, despite a significant increase in the body weight of these animals. These results suggest that a decrease in energy expenditure may be associated with the Olz-induced weight gain with the 3 mg/kg dose [33]. Coinciding with previous studies [32], [34], our findings report a positive correlation between body weight gain and visceral fat deposition despite Olz, OlzEt and OlzHomo inducing low visceral fat deposition. Both peripheral and central factors may be involved in Olz-induced weight gain and adiposity, nevertheless the exact mechanisms by which this drug causes metabolic adverse side effects still remains elusive [33]. The fat deposition may be associated with the increase in body weight or be due to the direct effects of Olz on adipose tissue [35], [36]. Studies reporting Olz-mediated peripheral adipogenesis in the 3T3-L1 cell model showed an over-expression of *fatty acid synthase* and *adiponectin* genes [37]. In accordance with this *in vitro* adipogenesis result and with previous animal and clinical studies [34], [38], our findings support the effect of Olz administration on increasing plasma adiponectin levels. However, our data may appear counterintuitive given that other studies reported a reduction of adiponectin in obesity [39], [40]. The correlation between plasma leptin levels and visceral adiposity found in this study suggested that leptin may be a useful indicator of fat mass deposition induced by Olz. Interestingly, we found plasma leptin levels in animals treated with OlzHomo were significantly reduced, which may confirm the preventative effect of OlzHomo on visceral adiposity. A similar outcome was found in the leptin levels of female rats treated with ziprasidone, which has a low effect on weight gain and fat deposition [41]. Numerous studies have shown that Olz-induced weight gain is associated with elevated leptin levels in schizophrenia patients [42], [43], [44], [45]. Since we have observed a link between enhanced adiposity and leptin levels, the lack of a significant effect of Olz treatment on leptin levels in our study may appear surprising. However, our treatment duration was subchronic (5 weeks), which can explain the discrepancy of our data with chronic studies performed with schizophrenia patients [42], [43], [44], [45]. Our study also showed a marked reduction in insulin secretion in the 6 mg/kg Olz treatment group with no significant change in the 3 mg/kg Olz, OlzEt, and OlzHomo-treated animals as illustrated in Figure 3. These results support some recent findings that short term treatment with Olz decreased insulin levels in rats and schizophrenia patients [28], [46]. On the other hand, an extensive amount of literature has reported increased insulin levels following chronic treatment with Olz, particularly in patients who gained a significant amount of weight [47], [48], [49], [50]. However, as a result of increasing weight gain, chronic administration of Olz can lead to compensatory hyperinsulinemia and insulin resistance, which are commonly observed in clinical cases. The direct antagonistic effect of Olz delivered at a high dose (6 mg/kg in rats represents around double the dose for treatment in humans) on the muscarinic M_3_ receptors in the pancreatic β-cells which regulate insulin secretion may contribute to the reduction in plasma insulin concentrations observed in our study [52].

With regards to the levels of plasma glucose, our results did not show any significant difference between the different treatment groups (Olz, OlzEt and OlzHomo) compared to the controls, which is in accordance with results from recent clinical studies [71]. Since blood glucose levels depend not only on insulin secretion, but also on tissue insulin utilisation, our results suggest that there is no insulin resistance at the early stage of olanzapine treatment in our study. Although weight gain was significant in rats treated with Olz *vs.* vehicle rats, these animals seemed to remain insulin sensitive. This is supported by our results showing no significant difference in the lipid profiles performed in the Olz treated rats compared to the vehicle rats.

The affinities of atypical antipsychotics for the 5HT_2A_, 5HT_2C_, 5HT_6_, α_1A_ and H_1_ receptors and their obesogenic effects has been repeatedly reported in various studies [2], [15], [16], [17]. Particular emphasis was placed on the ability of antipsychotics to block the H_1_ receptors [19], [20]. The blockade of the H_1_ receptors is directly involved in the activation of the hypothalamic AMPK signalling pathway, which stimulates food intake and positive energy balance and reverses the anorexigenic effect of leptin [18]. This study supports the potential role of H_1_ receptor affinity in antipsychotic-induced weight gain and fat deposition. As shown in our previous report [27], OlzEt and OlzHomo have a lower affinity for binding to the H_1_ receptors (*K*_i_ = 1.95, and *K*_i_ = 13.63, respectively) compared to that of Olz (*K*_i_ = 0.13). Therefore, the pronouced antagonism of OlzEt and OlzHomo at the H_1_ receptors may be responsible for their significantly attenuated propensity to induce weight gain and metabolic dysfunction, which are associated with Olz treatment. Furthermore, corresponding with previous reports, the present study demonstrated a significant negative correlation between hypothalamic H_1_ receptor density and weight gain and accumulative fat mass in rats. H_1_ receptor density in the hypothalamus has been markedly reduced following chronic treatment with Olz (at both 3 mg/kg and 6 mg/kg doses) but not with OlzEt and OlzHomo, supporting the importance of the H_1_ receptor in Olz-induced obesogenic side effects. In agreement with our observation, the down-regulation of H_1_ receptor expression has been previously reported in the hypothalamic nuclei of rats treated with Olz [33]. In addition, the nonsignificant alterations of the H_1_ receptor density following OlzEt and OlzHomo treatments may explain the lack of orexigenic effects of these two compounds in the treated rats. Thus, the involvement of the H_1_ receptors in Olz-induced obesity and fat deposition might be closely related.

In general, while our study confirmed the effect of Olz administration on metabolic alterations in rats, we showed that OlzEt and OlzHomo administrations did not induce either enhancing effects on body weight and food intake or detrimental consequences on fat deposition and metabolism. Our findings appear to have reasonable predictive validity for different aspects of Olz-induced weight gain/adiposity and metabolic abnormalities which mimic the clinical situation.

In conclusion, our findings confirmed the obesogenic effect of Olz administration, coupled with the down-regulation of the H_1_ receptors in the hypothalamus. Our results showed that both OlzEt and OlzHomo appear to be promising new candidate compounds which did not result in weight gain, visceral fat deposition and metabolic dysfunction. However, based on the pharmacological proprieties alone, OlzEt presented with a similar profile to Olz during behavioural assessment in the open-field test with regards to blocking PCP-induced hyperactivities. These findings suggest that the long lasting down-regulation of D_2_ and 5-HT_2A_ receptors induced by sub-chronic Olz and OlzEt treatment may play a part in blocking PCP-induced behaviours. The fact that OlzHomo had a reduced capacity to inhibit PCP-induced behaviours could also be explained by its lower affinity for the D_2_ and 5HT_2A_ receptors in the brain compared to that of Olz and OlzEt. Therefore, if the OlzHomo regime was to be delivered at a higher dose than that of Olz and OlzEt, then the therapeutic effectiveness of OlzHomo may be increased. Given the limitations associated with animal models, we suggest that the present results be taken with caution. Only further behavioural studies and clinical trials will reveal the predictive validity of this preclinical model for the therapeutic efficacy and metabolic side effects of these two compounds.

## Supporting Information

Table S1 **Correlation and regression analysis.** Pearson's correlation tests for radioligand receptor binding, metabolic and hormonal parameters in female Sprague Dawley rats following 5 weeks treatment with Olz, OlzEt, OlzHomo (3 mg/kg or 6 mg/kg), or vehicle (Control).
(PDF)
