# Supplementary material for: In Vivo Pharmacological Evaluations of Novel Olanzapine Analogues in Rats: A Potential New Avenue for the Treatment of Schizophrenia
Source: PLoS One. 2013 Dec 11;8(12):e80979. doi: 10.1371/journal.pone.0080979 (PMC3859487; doi:10.1371/journal.pone.0080979)
Supplement: Table S1 — Correlation and regression analysis. Pearson's correlation tests for radioligand receptor binding, metabolic and hormonal parameters in female Sprague Dawley rats following 5 weeks treatment with Olz, OlzEt, OlzHomo (3 mg/kg or 6 mg/kg), or vehicle (Control). (PDF) [file pone.0080979.s001.pdf]

Table S1 Results of correlation and regression analysis

		r	*P*	r^2	*P*
Total body weight gain	Total food intake	0.48	0.000	0.197	0.001
Total body weight gain	Total white fat	0.42	0.002	0.170	0.002
Total body weight gain	H_1 receptor density	- 0.59	0.001	0.343	0.001
Total body weight gain	Insulin	- 0.310	0.036		
Total body weight gain	Adiponectin	0.63	0.000	0.394	0.001
Total food intake	Insulin	- 0.46	0.002	0.297	0.001
Total food intake	Leptin	0.36	0.020	0.223	0.002
Total food intake	Adiponectin	0.46	0.001	0.161	0.005
Total white fat	Adiponectin	0.29	0.041		
Total white fat	H_1 receptor density	-0.61	0.001	0.375	0.001
Total white fat	Insulin	0.34	0.019	0.116	0.019
Total white fat	Food	0.59	0.000	0.329	0.001
Total white fat	Leptin	0.344	0.026		
